# Mediterranean Diet Adherence and Risk of Depressive Symptomatology in a French Population-Based Cohort of Older Adults

**DOI:** 10.3390/nu14194121

**Published:** 2022-10-04

**Authors:** Jeanne Bardinet, Virginie Chuy, Isabelle Carriere, Cédric Galéra, Camille Pouchieu, Cécilia Samieri, Catherine Helmer, Audrey Cougnard-Grégoire, Catherine Féart

**Affiliations:** 1 Univ of Bordeaux, INSERM, BPH, UMR1219, F-33000 Bordeaux, France; 2 Activ’Inside, F-33750 Beychac-et-Caillau, France; 3 CHU de Bordeaux, Pôle de Médecine et Chirurgie Bucco-Dentaire, F-33000 Bordeaux, France; 4 Institut for Neurosciences of Montpellier INM, University Montpellier, INSERM, F-34091 Montpellier, France; 5 Centre Hospitalier Perrens, F-33000 Bordeaux, France; 6 Clinical and Epidemiological Research Unit, INSERM CIC1401, F-33000 Bordeaux, France

**Keywords:** Mediterranean diet, depressive symptomatology, older adults, Three-City, cohort

## Abstract

Several foods from the Mediterranean Diet (MeDi) have already been characterized as beneficial for depression risk, while studies focusing on adherence to the overall MeDi are lacking among older adults at higher risk of depression. The aim of this study was to assess the association between MeDi adherence and the risk of depressive symptomatology (DS) in an older French cohort followed for 15 years. Participants from the Three-City Bordeaux cohort answered a food frequency questionnaire used to assess their MeDi adherence. The Center for Epidemiologic Studies Depression (CES-D) scale score of 16 or greater and/or use of antidepressant treatment ascertained at each visit defined incident DS. Random-effect logistic regression models were adjusted for potential confounders. Among 1018 participants, aged 75.6 years (SD 4.8 years) on average at baseline, 400 incident cases of DS were identified during the follow-up. Only when restricting the definition of DS to a CES-D score ≥ 16 was a borderline-significant trend towards a benefit of greater adherence to the MeDi with reduced odds of DS found (*p*-value = 0.053). In this large sample of older French adults, a potential benefit of greater adherence to the MeDi regarding the risk of DS would depend on the definition of DS.

## 1. Introduction

In most developed countries, the population is aging, leading to the increase of chronic diseases, notably among the oldest people. For instance, prevalence of disability, loss of autonomy, and also cognitive and mental disorders have increased [1,2]. Mental disorders are currently a major public health concern, and among them, depression represents the leading cause of individual daily life and socioeconomic consequences [2]. Depression is estimated to affect more than 280 million people worldwide and older adults seem more prone to it, with prevalence estimated at around 6% [2,3]. In addition, one third of people are considered antidepressant-resistant in the general population [4], a figure that reaches one in two older adults [5]. In older age, risk factors for depression are multiple, encompassing physiological and social risk factors (such as chronic diseases, loss of autonomy, widowing…) and modifiable lifelong environmental factors, which could be leveraged for prevention. For instance, Worrall et al. reported that favorable social or family support, better self-rated health, and higher physical activity were associated with a lower risk of depression [6]. Interestingly, a high-quality diet may be an additional protective factor [7]. Observational studies reported higher prevalence rates of mental disorders in Northern European countries than in Mediterranean countries, which may be partly explained by geographical lifestyle and dietary habits [8,9]. Indeed, as part of a healthy lifestyle, the Mediterranean Diet (MeDi), characterized by a high intake of fruits, vegetables, legumes, cereals, fish and olive oil, a moderate intake of dairy products and alcohol, and a low intake of meat, is widely recognized as a healthy diet regarding overall mortality, cardiovascular diseases, cancers, an also mental disorders [10,11,12,13,14,15,16]. Several studies including reviews and meta-analyses have already reported that higher adherence to the MeDi was associated with a reduced risk of depression in population-based samples [10,12,17,18]. Two recent additional longitudinal studies have specifically examined this relationship among older populations and reported benefits of a greater MeDi adherence on the risk of depression [19,20]. However, combined with a third study on the oldest old [21], a recent meta-analysis failed to confirm such results, concluding there was no difference between the degree of adherence to the MeDi and the incidence of depression among older adults [22]. These three latter studies were prospective observational cohorts focusing on people aged 50 years and over and followed for up to 3–12 years. They originated from Australia and the United States, two countries far from the Mediterranean Basin, which could question the external validity of the results. To our knowledge, no longitudinal studies have examined the MeDi–depressive symptoms relationship among older adults living near the Mediterranean Basin, though likely highly traditional MeDi adherents. Altogether, we hypothesized the MeDi could be considered a promising lifestyle for decreasing the risk of depressive symptomatology (DS) in an older population living around the Mediterranean Basin. The main objective of the present study was to assess the association between MeDi adherence and the incidence of DS over a period of 15 years among older French adults.

## 2. Materials and Methods

### 2.1. Population

The study population was part of the Three-City (3C) cohort, a prospective population-based study started in 1999 in France with 9294 inhabitants of 3 municipalities (Bordeaux, Dijon, and Montpellier). The initial objective of this study was to examine the association between vascular risk factors and dementia. Recruitment was performed by random selection from the electoral rolls, and to be included, the participants had to live at home in one of the three cities or their suburbs and to be aged 65+. Participants were followed up every two to three years until 2018 (up to 7 waves). The Advisory Committee for the Protection of Persons Participating in Biomedical Research of the Centre Hospitalier Universitaire of Kremlin-Bicêtre approved the study protocol and each participant in the study signed a free and informed consent form [23]. The entire protocol of the 3C cohort is described elsewhere [14].

### 2.2. Study Sample

The study sample was restricted to the 3C Bordeaux cohort, where detailed nutritional data were collected in 2001–2002, considered as the baseline of the present analysis. The participants included in the present study were free of DS at the time of the dietary survey (baseline for the present study) and 2 years earlier (3C recruitment), as ascertained by their drug consumption and by the Center for Epidemiologic Studies Depression (CES-D) scale (detailed further). Finally, included participants were not demented at the time of 3C inclusion.

### 2.3. Adherence to the Mediterranean Diet

The dietary survey included a 24 h dietary recall and a food frequency questionnaire (FFQ) administered at home by dieticians. Adherence to the MeDi was assessed once from the FFQ, which recorded 148 foods and beverages, and the use of the MeDi-Lite score proposed by Sofi et al. [24]. As previously described, this score was computed using the frequency of consumption of 9 food groups characterizing the traditional MeDi [25]. Higher consumption of fruit, vegetables, legumes, cereals, fish and olive oil, moderate consumption of dairy products and alcohol, and low consumption of meat were considered beneficial. Sofi et al. determined the thresholds used for the attribution of points based on the frequency of consumption of each food group from those reported in the literature for the computation of the MeDi-Lite score (Supplementary Materials, Table S1) [24]. Then, in the present study, the score was divided into tertiles to define three degrees of adherence to the MeDi: “low”, “moderate”, and “high”.

### 2.4. Depressive Symptomatology

DS was assessed at each wave by a neuropsychologist during a face-to-face interview, using the CES-D scale, a validated 20-item scale [26,27]. The total CES-D score ranged from 0 to 60 according to the frequency (from “never” to “always,” rated from 0 to 3) of the depressive symptoms felt during the previous week. Scores of 16 or greater confirmed DS, as well as missing data due to severe depression, as ascertained by the neuropsychologist in charge of the survey. Any use of antidepressant treatment was recorded and its pharmacology class was identified according to the World Health Organization’s Anatomical Therapeutic Chemical corresponding classification “N06A” [28]. Thus, at each follow-up wave, DS was defined combining the CES-D score (i.e., ≥16) and/or the use of antidepressant treatment: this definition combined participants (i) with high CES-D score without recourse to medication, OR (ii) with low CES-D score and antidepressant use being efficient (therapeutic success), and OR (iii) participants with high CES-D score and antidepressant use later being inefficient (antidepressant nonresponders).

### 2.5. Covariates

From the dataset, we retained the following data for the description of the studied sample: sex, age, living conditions (living alone, in couple and cohabitation), educational level (no study or elementary, secondary, high school and university), monthly income (<EUR1500, 1500–2250, ≥2250, and refused to answer), tobacco consumption (number of pack-years), body mass index (BMI; measured and expressed as <25, 25–30 and ≥30 kg/m^2^), practice of regular physical activity (yes or no), total energy intake (in kcal/day) and Mini Mental State Examination (MMSE) score. Multimorbidity (yes or no) was summarized as ≥2 health disorders among hypertension, diabetes, hypercholesterolemia, angina, cardiac rhythm disorders, arteritis, cardiac failure, myocardial infarction, hospitalization for stroke, asthma, Parkinson’s disease, dyspnea, osteoporosis, dementia, and cancer [29]. All these health disorders were self-reported at baseline of the present study, except for dementia, which was clinically diagnosed. Among these covariates, the most relevant potential confounders were identified from information found in the literature and with a directed acyclic graph (DAG).

### 2.6. Statistical Analysis

All statistical analyses were achieved using RStudio Software (Allaire J.J., South Bend, USA, version 1.3.1093) and statistical significance was set at *p* < 0.05. We firstly described and compared the characteristics of the included participants according to the degree of adherence to the MeDi from MeDi-Lite score using chi-squared tests for qualitative variables and analysis of variance for quantitative variables (nonparametric ones for those with asymmetric distribution). The weekly food group consumption used to define the MeDi-Lite score was also described according to the degree of adherence to the MeDi.

### 2.7. Association between MeDi Adherence and Risk of DS

Random-effect logistic regression was performed to examine the association between MeDi adherence (the reference category was chosen as the lowest adherence tertile) and the risk of DS over time adjusted for the baseline value of all retained potential confounders: age (time scale in the model), sex, educational level, living conditions, physical activity, tobacco consumption, BMI, total energy intake, and multimorbidity. Each model included time, MeDi adherence and all the selected covariates. The interaction between MeDi adherence and time was not significant and therefore not introduced in the model. The group probabilities of DS (in logit scale) vary thus in parallel through time and the estimated odds ratios correspond to the constant differences through time between the MeDi groups. The random-effect logistic model uses all observed data to estimate the model parameters, and thus was appropriate for the longitudinal data. Indeed, this method takes into account all individuals, even if they are followed up only once over time, in order not to lose information. In this analysis, DS was the binary response (yes/no) and two types of random effects were introduced into the model: a random intercept, assuming heterogeneity between individuals at the first evaluation, and a random slope, assuming variability over time. In order to estimate the risk of developing DS as a function of age, the function of time corresponded to the time elapsed since the age of 65 years (inclusion criteria in the 3C cohort).

### 2.8. Additional Analyses

#### 2.8.1. CES-D Score ≥ 16

First, considering that antidepressant treatment may be prescribed for other indications, the DS was also defined only as having a CES-D score ≥ 16, irrespective of antidepressant use.

#### 2.8.2. Sex-Specific Cutoffs for Identifying DS

Subsequently, DS was defined using validated sex-specific French cutoffs (i.e., CES-D score ≥ 17 for men and ≥23 for women) and/or antidepressant treatment use [30].

#### 2.8.3. Mediterranean Diet Adherence Assessed by Two Alternative Scores

Adherence to the MeDi was also estimated using two other approaches: the Mediterranean Diet Score (MDS) proposed by Trichopoulou et al. [31] (ranged from 0 to 9) and the MedDietScore proposed by Panagiotakos et al. [32] (ranged from 0 to 55). The computation of these scores has been detailed in Supplementary Materials (Tables S2 and S3).

### 2.9. Missing Data

Some covariates had missing data: to keep the maximum of information, we ran a multiple imputation by chained equations using the mice packages [33], an elaborate strategy limiting the bias introduced by the missing data under certain conditions (assumption of missing at random data) [34]. Each variable is associated with an imputation model conditionally to other selected variables of the dataset. Ten imputed datasets were generated and we combined the results from analyses on each of them to obtain the summary fixed effects. Among selected potential confounders, tobacco consumption had 14 missing values (1.4%), BMI had 10 missing values (1.0%), total energy intake had 3 missing values (0.3%) and regular physical activity had 152 missing values (14.9%).

## 3. Results

### 3.1. Sample Selection

**Figure 1:**
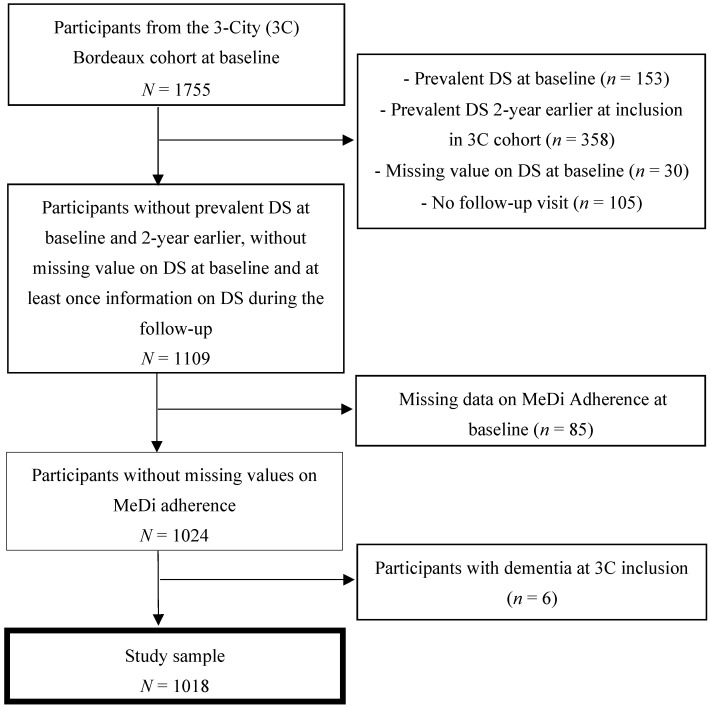
Flowchart of study of the association between adherence to the Mediterranean diet and the incidence of depressive symptomatology in a population aged 65 years and over. Three-City Bordeaux cohort, 2001–2017; DS = depressive symptomatology; MeDi = Mediterranean diet.

The 3C Bordeaux cohort was composed of 1755 individuals at baseline of the present analysis (first year of follow-up in 3C). We excluded participants with: (i) prevalent DS at study baseline (*n* = 153) or 2-year earlier at inclusion in the 3C cohort (*n* = 358), (ii) missing data for DS at baseline (*n* = 30) or with no follow-up visit (*n* = 105), (iii) missing data on MeDi adherence (*n* = 85), and (iv) demented at 3C inclusion (*n* = 6), leading to a study sample of 1018 individuals (Figure 1). The median follow-up time was 11.5 years (minimum 1.6 and maximum 16.3 years). Individuals had on average 4 follow-up visits and 372 participants were present at the last visit, while 522 died during the follow-up.

### 3.2. Descriptive Characteristics of the Study Sample

The study sample included 57.2% women with a mean age of 75.6 years (standard deviation (SD): 4.8 years) at baseline (Table 1). More than half the participants (58.9%) lived as a couple. Thirty-two percent of the studied sample were highly educated (university level) and 30.6% declared a monthly income of EUR2250 or more. Participants had smoked on average 9.5 (SD: 18.1) pack-years, 45.9% had a BMI between 25 and 30 kg/m^2^ and more than half of them (53.7%) were not physically active. The participants consumed on average 1741 kcal/day (SD: 540) and had an MMSE score of 27.8 points on average. More than half the participants (54.0%) were concerned by multimorbidity and the mean CES-D score was 4.9 (± 4.0) at baseline.

### 3.3. Adherence to the Mediterranean Diet

MeDi-Lite score was 10 on average (minimum 4 and maximum 16) and the lowest adherents (*n* = 257, 25.3%) had a score lower than or equal to 9, those with moderate adherence (*n* = 389, 38.2%) between 10 and 11, and the highest adherents (*n* = 372, 36.5%) equal to or greater than 12. Women were less often low MeDi-Lite adherents than men. At baseline, individuals with the lowest MeDi-Lite score smoked more and were less often concerned by multimorbidity than the other adherents were. Regardless of the degree of adherence to the MeDi, participants exhibited on average similar ages, living conditions, educational levels, monthly incomes, BMI, physical activity, total energy intake, cognitive performances on MMSE, and CES-D scores at baseline (Table 1).

The description of weekly food group consumption according to the MeDi-Lite score categories showed that the highest adherents had a significantly higher consumption of fruits, vegetables, legumes, cereals, and fish, more regular use of olive oil, and lower consumption of meat and dairy products, as expected by the score computation, and they had lower alcohol consumption (Table 2).

### 3.4. Incidence of DS over Time

A total of 400 incident cases of DS (39.3%) were identified during the 15-year follow-up. Among them, 218 participants (54.5%) exhibited a CES-D score ≥ 16 at least once over time, 103 participants (25.7%) had antidepressant treatment prescribed at least once over time and 79 participants (19.8%) had both CES-D score ≥ 16 and antidepressant treatment prescribed at least once over time.

### 3.5. Association between MeDi Adherence and Risk of DS

Greater MeDi adherence, assessed by the MeDi-Lite score, was not significantly associated with reduced odds of DS over time after controlling for age (time scale in the model), sex, educational level, living conditions, physical activity, tobacco consumption, BMI, total energy intake and multimorbidity (odds ratio (OR) = 0.82, 95% confidence interval (CI) [0.52;1.30] and OR = 0.72, 95% CI [0.45;1.16] for the moderate and for the highest MeDi adherents, respectively, compared with the lowest MeDi adherents, *p*-value for trend = 0.404) (Table 3). However, we observed that being a woman, living in a couple or in cohabitation (vs alone), being older and concerned by multimorbidity were significantly and independently associated with an increased risk of DS in the multivariate analysis (OR = 1.94, 95% CI [1.22; 3.08], *p*-value = 0.005 for women vs. men; OR = 1.71, 95% CI [1.14; 2.56] and OR = 2.23, 95% CI [1.02; 4.88], for living in couple and in cohabitation, respectively, vs. alone, *p*-value for trend = 0.017 and OR = 1.79, 95% CI [1.24; 2.58], *p*-value = 0.002 for individual concerned by multimorbidity vs. not).

### 3.6. Additional Analyses

#### 3.6.1. CES-D Score ≥ 16

When considering the definition of DS restricted on the CES-D scores (i.e., ≥16), 297 participants (29.2%) developed a DS during the follow-up. Using this definition of DS, there was a trend towards a greater adherence to the MeDi being associated with reduced odds of DS, but the association was borderline significant (OR = 0.72, 05% CI [0.46; 1.11] and OR = 0.57, 95% CI [0.36; 0.90], respectively, for moderate and high adherents compared with low adherents; *p*-value for trend = 0.053) (Table 4).

#### 3.6.2. Sex-Specific Cutoffs for Identifying DS

Using the French sex-specific cutoffs (i.e., CES-D score ≥ 17 for men and ≥ 23 for women) and/or antidepressant use for defining DS, we identified 321 incident cases (31.5%) during follow-up. The association between adherence to the MeDi and the incidence of DS with sex-specific cutoffs was not statistically significant after controlling for all selected confounders (OR = 0.95, 95% CI [0.48; 1.88] and OR = 0.76, 95% CI [0.38; 1.53] for moderate and high adherents, respectively, compared with low adherents; *p*-value for trend = 0.678) (Supplementary Materials, Table S4).

#### 3.6.3. Mediterranean Diet Adherence Assessed with Two Alternative Scores

Then, we used two alternative scores to assess MeDi adherence: the MDS and the MedDietScore. The association between the adherence to the MeDi according to the MDS or the MedDietScore and the incidence of DS (defined as CES-D ≥ 16 and/or antidepressant treatment use) was not statistically significant after adjustment for all confounders (*p*-value for trend = 0.301 and 0.560, respectively) (Table 5).

## 4. Discussion

In this large prospective sample of older French adults followed for up to 15 years, no significant association was observed between MeDi adherence and the risk of DS using different approaches to assess MeDi adherence. However, when DS was restricted to individuals with a CES-D score ≥ 16 irrespective of medication use, a trend was observed for an inverse association between a greater adherence to the MeDi and the odds of DS. Thus, a healthy diet such as the MeDi might be a promising approach, although probably not sufficient alone for older adults.

Overall, previous studies focusing on the relationship between MeDi adherence and risk for depression in the general adult population have reported inconsistent results, as summarized in two recent meta-analyses (seven joint studies). In the first meta-analysis [10], the combined seven prospective cohorts (of more than 50,000 adults from France, the USA, Australia, and Spain) reported a lack of association between MeDi adherence assessed by the MDS or the MedDietScore and the risk of DS assessed by different depression scales, medical diagnosis or use of antidepressant treatment. In the second meta-analysis [18], when restricting results to the combination of the four retained longitudinal studies (all already included in the previous described meta-analysis), the highest adherence to the MeDi (assessed with MDS or rMDS, which differed only by fat component) was associated with a significant 33% reduced odds of DS compared to the lowest adherence (overall OR = 0.67, 95% CI [0.55; 0.82]). Of note, only two studies included in this meta-analysis were implemented near the Mediterranean Basin and none was focused on older adults. A single French study, focusing on adults involved in the SU.VI.MAX randomized trial, reported no association between MeDi adherence (assessed with the MDS) and the risk of DS [35]. In the second study, from the SUN cohort on Spanish students, DS was assessed using self-reported diagnosis and antidepressant treatment. High adherence to the MeDi (also assessed with the MDS) was associated with a significantly reduced risk of DS compared to the lowest adherence with a significant dose–response relationship [36]. While these European studies were close to the present ones regarding location and therefore with consumption close to a South European lifestyle, results were not strictly comparable because of the heterogeneity regarding the age of participants involved. Altogether, these results, implemented near the Mediterranean area, suggest a promising protective relationship between higher MeDi adherence and the risk of DS without confirming it at older ages. Finally, MeDi adherence is also part of a healthier lifestyle in general, especially in countries far from the Mediterranean Basin, which can favorably affect mental health and mood.

Inconsistent results observed worldwide could be explained in part by the difficulty in identifying DS among the community of the oldest old. Indeed, the increase in age and in age-related health disorders could be accompanied by subtle changes in mood, while unnoticed or considered classical traits of aging, and on the other hand, depressive disorders could be expressed as atypical form in older adults (i.e., aggressive or addictive behaviors, anxiety and personality disorders), leading to potential misclassification bias [37,38]. Moreover, depression is multidimensional and involves many individual and societal factors, especially in older people (i.e., social isolation, life event, health issues, economic changes…) against which the adoption of a healthy diet as a single preventive dimension seems insufficient to guarantee a better mood [39]. Indeed, the MeDi would be inefficient at older ages (mean age of the present study sample was 75.6 years, with a follow-up of up to 16.3 years) or may be less effective in older adults than antidepressant treatment to postpone depression/prevent the development of depressive symptoms in a complex context, including polypathology during aging and/or environmentally deleterious conditions accompanying aging (widowhood for instance). Interestingly, while fruit and vegetables, as food sources of antioxidants [40,41], and fish, as a source of long-chain omega-3 fatty acids, are part of MeDi components that would reduce the risk of depression [42], other interesting foods or nutrients not part of the MeDi would potentially contribute to beneficial or deleterious associations with the risk of DS. For instance, the consumption of coffee or tea are notably rich providers of polyphenols, with potential neuroprotection due to their antioxidant and anti-inflammatory properties [41]. On the other hand, sweetened beverages, salt, or other fats than olive oil, involved in increased inflammation and oxidative stress, would contribute to the pathogenesis of depression [43,44,45]. Another mechanism involved in this relationship concerns an essential amino acid, tryptophan, supplied by a variety of foods (including peanuts, oats and bananas), which represent a good avenue against depression due to its conversion in serotonin, the target of a majority of antidepressant treatments [44]. Finally, the diet–depression relationship is underpinned by numerous correlated mechanisms including neurogenesis, epigenetic, hypothalamus–pituitary–adrenal axis, and mitochondrial dysfunction in addition to aging, while we cannot ascertain them [44].

Regarding the present study, it is important to point out some limits. In the 3C cohort, the dietary survey was administered only in the 3C Bordeaux subsample, which may induce a selection bias, though reduced by the representativeness of the 3C Bordeaux sample during the follow-up [46]. We assumed the stability of food behaviors over time, which is acceptable for the whole group [47], but we cannot totally exclude a change in dietary habits of a specific individual over time (for clinical reasons, change in living conditions, diseases requiring a specific diet…), although we cannot ascertain specifically these changes. Moreover, the computation of scores to assess adherence to the MeDi caused several dietary patterns to coexist in a single category of MeDi adherents, as already pointed out [48]. Regarding DS, the CES-D scale is not a clinical diagnostic tool, though validated and largely used in epidemiological studies [10,18,19,20]. The inclusion of antidepressant treatment in the definition of the outcome is also debatable, since it leads to a heterogeneous group of participants with incident DS efficiently treated or not and because these treatments being not only prescribed for depression [49]. This led us to test the robustness of our results in an additional analysis not including antidepressant treatment in the definition of DS, which potentially minimized the misclassification bias. Moreover, alcohol consumption is linked to depression and could have been considered as a main confounder as in other studies, but as one of nine food groups are part of the MeDi, it could have led to an overadjustment and we chose to not include it in the present study. A final point concerns the potential reverse-causality bias, in part limited thanks to the long follow-up of the present analysis and by the exclusion of participants with DS at baseline and at the previous examination. However, we cannot exclude that greater MeDi adherents at baseline, who reported higher multimorbidity than low MeDi adherents, have been engaged in this healthy dietary lifestyle as an additional management of their diseases. Despite these limitations, it is also relevant to highlight all the strengths of the present study. The large sample of more than 1000 older adults, the long duration of 15 years, the interviews using standardized questionnaires using validated tools (CES-D and FFQ) performed by trained professionals, and the consideration of main confounders (although residual confounders may persist in observational studies) are the main strengths of the present analysis. In addition, the innovative statistical approach allowed us to use all information from repeated measures and consider potential changesin depression over time.

## 5. Conclusions

No association was observed between MeDi adherence and the risk of DS in a French population-based sample aged 65 years and over, followed for up to 15 years. However, a trend suggesting a potential benefit of a greater adherence to the MeDi on the basis of reduced odds of DS when restricting the definition of DS to the CES-D scores has been observed. This study adds to the available literature, specifically on older people, and suggests that adopting healthy dietary patterns appears insufficient to maintain satisfying mental health in older adults. Future studies are warranted to explore this complex relation and to better understand the underlying mechanisms.

## Figures and Tables

**Table 1:** Description of sociodemographic and lifestyle characteristics of the study sample according to adherence to the Mediterranean diet from the MeDi-Lite score, from the Three-City Bordeaux cohort, 2001–2017. (*N* = 1018).

	Mediterranean Diet Adherence ^1^				
	Overall (*n* = 1018)	Low Score ≤ 9 (*n* = 257)	Moderate Score = [10, 11] (*n* = 389)	High Score ≥ 12 (*n* = 372)	*p*-Value ^4^
	*N* (%)/ Mean ± SD	*N* (%)/ Mean ± SD	*N* (%)/ Mean ± SD	*N* (%)/ Mean ± SD	
Women	582 (57.2)	130 (50.6)	239 (61.4)	213 (57.3)	0.024
Age (years)	75.6 ± 4.8	75.5 ± 5.0	75.7 ± 4.7	75.7 ± 4.8	0.543
Living conditions					0.521
- Alone	358 (35.2)	100 (38.9)	131 (33.7)	127 (34.1)	
- In couple	600 (58.9)	140 (54.5)	234 (60.2)	226 (60.8)	
- Cohabitation (family or not)	60 (5.9)	17 (6.6)	24 (6.2)	19 (5.1)	
Educational level					0.086
- No study or elementary	296 (29.1)	83 (32.3)	112 (28.8)	101 (27.2)	
- Secondary	280 (27.5)	84 (32.7)	95 (24.4)	101 (27.2)	
- High school	112 (11.0)	22 (8.6)	48 (12.3)	42 (11.3)	
- University	330 (32.4)	68 (26.5)	134 (34.4)	128 (34.4)	
Monthly income, EUR ^*a*^					0.129
- <1500	373 (36.6)	113 (44.0)	136 (35.0)	124 (33.3)	
- 1500–2250	267 (26.2)	62 (24.1)	109 (28.0)	96 (25.8)	
- ≥2250	311 (30.6)	69 (26.8)	116 (29.8)	126 (33.9)	
- Refused to answer	67 (6.6)	13 (5.1)	28 (7.2)	26 (7.0)	
Number of pack-years smoked ^*b*^	9.5 ± 18.1	12.8 ± 22.0	9.0 ± 17.0	7.7 ± 15.9	0.001
Body mass index (in kg/m^2^) ^*c*^					0.208
- <25	379 (37.6)	90 (35.6)	137 (35.6)	152 (41.1)	
- 25–30	463 (45.9)	113 (44.7)	182 (47.3)	168 (45.4)	
- ≥30	166 (16.5)	50 (19.8)	66 (17.1)	50 (13.5)	
Regular physical activity ^*d*^	319 (31.3)	62 (24.1)	133 (34.2)	124 (33.3)	0.023
Total energy intake (kcal/day) ^*e*^	1741 ± 540	1752 ± 580	1729 ± 538	1746 ± 513	0.931
Mini Mental State Examination score ^*f*^	27.8 ± 1.9	27.7 ± 2.0	27.8 ± 1.8	27.8 ± 2.0	0.508
Multimorbidity ^2^	550 (54.0)	120 (46.7)	216 (55.5)	214 (57.5)	0.021
CES-D score at baseline	4.9 ± 4.0	4.9 ± 3.9	4.8 ± 4.1	5.0 ± 3.9	
DS ^3^ during the follow-up	400 (39.3)	104 (40.5)	155 (39.8)	141 (37.9)	0.779
- CES-D score ≥ 16	297 (29.2)	84 (32.7)	111 (28.5)	102 (27.4)	
- Antidepressant use	182 (17.9)	37 (14.4)	73 (18.8)	72 (19.4)	

SD = standard deviation; CES-D = Center for Epidemiologic Studies Depression; DS = depressive symptomatology. ^*a*^ Missing data for *n* = 7, ^*b*^ for *n* = 14, ^*c*^ for *n* = 10, ^*d*^ for *n* = 152, ^*e*^ for *n* = 3, ^*f*^ for *n* = 2. ^1^ MeDi-Lite score ranges from 0 to 18. ^2^ ≥2 Self-reported health disorders: hypertension, diabetes, hypercholesterolemia, angina, cardiac rhythm disorders, arteritis, cardiac failure, myocardial infarction, hospitalization for stroke, asthma, Parkinson’s disease, dyspnea, osteoporosis, dementia, and cancer. ^3^ DS = CES-D ≥ 16 and/or antidepressant treatment use. ^4^ *p*-value from chi-squared tests for qualitative variables and from analysis of variance for quantitative variables.

**Table 2:** Description of weekly food group consumption of the study sample according to adherence to the Mediterranean diet from MeDi-Lite scores (*N* = 1018).

		Mediterranean Diet Adherence ^1^		
Food Groups	Overall (*n* = 1018)	Low Score ≤ 9 (*n* = 257)	Moderate Score = [10, 11] (*n* = 389)	High Score ≥ 12 (*n* = 372)
	*N* (%) Mean ± SD	*N* (%) Mean ± SD	*N* (%) Mean ± SD	*N* (%) Mean ± SD
Fruits	13.6 ± 6.6	9.3 ± 6.5	13.8 ± 6.1	16.2 ± 5.6
Vegetables	19.5 ± 7.2	15.7 ± 6.8	19.5 ± 6.8	22.3 ± 6.6
Legumes	0.6 ± 0.7	0.5 ± 0.7	0.6 ± 0.7	0.8 ± 0.6
Cereals	22.3 ± 5.9	20.8 ± 6.9	22.2 ± 5.9	23.4 ± 4.9
Fish	3.0 ± 1.8	2.0 ± 1.4	3.0 ± 1.7	3.6 ± 1.8
Dairy products	16.0 ± 7.2	17.4 ± 7.4	15.7 ± 6.7	15.4 ± 7.5
Alcohol	11.1 ± 12.6	15.3 ± 16.4	10.6 ± 12.2	8.8 ± 8.8
Meat	4.9 ± 2.5	5.9 ± 2.9	4.8 ± 2.4	4.3 ± 1.9
Olive oil				
- Occasional use	393 (38.6)	175 (68.1)	164 (42.2)	54 (14.5)
- Frequent use	353 (34.7)	59 (23.0)	152 (39.1)	142 (38.2)
- Regular use	272 (26.7)	23 (8.9)	73 (18.8)	176 (47.3)

^1^ MeDi-Lite score ranges from 0 to 18.

**Table 3:** Association between Mediterranean diet adherence and the risk of depressive symptomatology (i.e., CES-D score ≥ 16 and/or antidepressant treatment) (*N* = 1018).

	Incident DS/Total	OR [95% CI] **	*p*-Value ***
Mediterranean diet adherence *			0.404
- Low adherence	104/257	1	
- Moderate adherence	155/389	0.82 [0.52; 1.30]	
- High adherence	141/372	0.72 [0.45; 1.16]	

DS = depressive symptomatology. * MeDi-Lite score = low adherence: score 0 to 9; moderate adherence: score 10 to 11; high adherence: score 12 to 18. ** Random-effect logistic regression model with a random intercept and a random slope adjusted for age (in the model time), sex, educational level, living conditions, physical activity, tobacco consumption, body mass index, total energy intake and multimorbidity. *** *p*-value of the log-likelihood ratio test.

**Table 4:** Association between Mediterranean diet adherence and the risk of depressive symptomatology (i.e., CES-D score ≥ 16) (*N* = 1018).

	Incident DS/Total	OR [95% CI] **	*p*-Value ***
Mediterranean diet adherence *			0.053
- Low adherence	84/257	1	
- Moderate adherence	111/389	0.72 [0.46; 1.11]	
- High adherence	102/372	0.57 [0.36; 0.90]	

DS = Depressive symptomatology. * MeDi-Lite score = low adherence: score 0 to 9; moderate adherence: score 10 to 11; high adherence: score 12 to 18. ** Random-effect logistic regression model with a random intercept and a random slope adjusted for age (in the model time), sex, educational level, living conditions, physical activity, tobacco consumption, body mass index, total energy intake and multimorbidity. *** *p*-value of the log-likelihood ratio test.

**Table 5:** Association between Mediterranean diet adherence and the risk of depressive symptomatology (i.e., CES-D score ≥ 16 and/or antidepressant treatment) (*N* = 1018).

	Incident DS/Total	OR [95% CI] **	*p*-Value ***
Mediterranean diet adherence *			
According to the MDS			0.301
- Low adherence	103/251	1	
- Moderate adherence	184/441	0.86 [0.55; 1.35]	
- High adherence	113/326	0.69 [0.42; 1.12]	
According to the MedDietScore			0.560
- Low adherence	128/332	1	
- Moderate adherence	151/373	0.87 [0.53; 1.44]	
- High adherence	121/313	0.75 [0.44; 1.27]	

DS = depressive symptomatology. * MDS: Mediterranean Diet Score by Trichopoulou et al. = low adherence: score 0 to 3; moderate adherence: score 4 to 5; high adherence: score 6 to 9. MedDietScore: Mediterranean Diet adherence by Panagiotakos et al. = low adherence: score 0 to 29; moderate adherence: score 30 to 32; high adherence: score 33 to 55. ** Random-effect logistic regression model with a random intercept and a random slope adjusted for age (in the model time), sex, educational level, living conditions, physical activity, tobacco consumption, body mass index, total energy intake and multimorbidity. *** *p*-value of the log-likelihood ratio test.

## Data Availability

Data described in the manuscript, code book, and analytic code will be made available upon request: http://www.three-city-study.com/ancillary-studies.php (accessed on 30 September 2022).

## Supplementary Materials

The following supporting information can be downloaded at: https://www.mdpi.com/article/10.3390/nu14194121/s1, e-Method S1: Adherence to the Mediterranean Diet through three different approaches: the MeDi-Lite score, the Mediterranean Diet Score MDS and the MedDietScore; Table S1: Adherence to the Mediterranean Diet according to the MeDi-Lite score (0–18) (Sofi et al.); Table S2: Adherence to the Mediterranean Diet according to the Mediterranean Diet Score MDS (0–9) (Trichopoulou et al.); Table S3: Adherence to the Mediterranean Diet according to the MedDietScore (0–55) (Panagiotakos et al.); Table S4: Association between Mediterranean Diet adherence and the risk of depressive symptomatology (i.e., CES-D score ≥ 17 for men and CES-D score ≥ 23 for women and/or antidepressant treatment).
