# Supplementary material for: Mediterranean Diet Adherence and Risk of Depressive Symptomatology in a French Population-Based Cohort of Older Adults
Source: Nutrients. 2022 Oct 4;14(19):4121. doi: 10.3390/nu14194121 (PMC9614601; doi:10.3390/nu14194121)
Supplement: Supplementary file 1 [file nutrients-14-04121-s001.zip › nutrients-1942640-SI.pdf]

Mediterranean Diet Adherence and Risk of Depressive Symptomatology in a French Population-Based Cohort of Older Adults

Jeanne Bardinet [1,2], Virginie Chuy [1,3], Isabelle Carriere [4], Cédric Galéra [1,5], Camille Pouchieu [2], Cécilia Samieri [1], Catherine Helmer [1,6], Audrey Cougnard-Grégoire [1] and Catherine Féart [1,*]

Supplementary Materials

List of Elements

e-Methods S1. Adherence to the Mediterranean Diet through three different approaches: the MEDI-LITE score, the Mediterranean Diet Score MDS and the MedDietScore.

Table S1. Adherence to the Mediterranean Diet according to the MEDI-LITE score (0-18) (Sofi *et al.)*

Table S2. Adherence to the Mediterranean Diet according to the Mediterranean Diet Score MDS (0-9) (Trichopoulou *et al.)*

Table S3. Adherence to the Mediterranean Diet according to the MedDietScore (0-55) (Panagiotakos *et al.)*

Table S4. Association between Mediterranean Diet adherence and the risk of depressive symptomatology (i.e. CES-D score ≥17 for men and CES-D score ≥23 for women and / or antidepressant treatment)

e-Methods S1. Adherence to the Mediterranean Diet through three different approaches: the MEDI-LITE score, the Mediterranean Diet Score MDS and the MedDietScore.

The MEDI-LITE Score (**Table S1**)

The MEDI-LITE score proposed by Sofi *et al.* was based on literature data to estimate the adherence to the Mediterranean Diet (MeDi) using the frequency of consumption of 9 food groups from the traditional MeDi (1). A high consumption of fruits, vegetables, legumes, cereals, fish and olive oil, a moderate consumption of dairy products and alcohol, and a low consumption of meat defined a high adherence to the MeDi in this score. Consumption frequency thresholds were estimated by the authors from literature and 0, 1 or 2 points were attributed for each food group, depending on the consumption of the food group in the diet, as detailed in **Table S1**. The obtained score summing the values of the 9 food groups indicated the degree of adherence to the MeDi, from 0 to 18 (the highest adherence).

The Mediterranean Diet Score MDS (**Table S2**)

The MDS proposed by Trichopoulou *et al*. was the first created to estimate the adherence to the MeDi (2). Currently, this score is composed by the same 9 food groups as for the MEDI-LITE score, except for olive oil: the MDS considered a ratio monounsaturated fatty acids (MUFA) to saturated fatty acids (SFA) instead of olive oil intake. For each food group, except for alcohol, a cutoff point is defined, based on the gender-specific median daily consumption frequencies of the study sample (**Table S2**). For food groups considered representative to the MeDi such as fruits, vegetables, cereals, legumes, fish and the ratio of MUFA to SFA, a value of 0 is assigned if the consumption of the individual is below the sex-specific median of the studied sample or a value of 1 if the consumption is above. For foods considered as detrimental, as meat, meat products and dairy products, the scoring is reversed and a value of 0 is given for an individual's consumption above the threshold and a value of 1 for an individual's consumption below it. With regard to alcohol consumption, 1 point is attributed for a man who consumed between 10 and 50 grams per day and between 5 and 25 grams per day for a woman ; in other cases (above or below these quantities), the value 0 is awarded to each participant. Here again, the obtained score summing the values of the 9 food groups indicates the degree of adherence to the MeDi, from 0 to 9 (the highest adherence).

The MedDietScore (**Table S3**)

The MedDiestScore from Panagiotakos *et al.* takes into account 11 food groups: potatoes and poultry in addition to the 9 food groups of the MEDI-LITE score (3). In addition, the weekly consumption frequency of each group is classified into 6 categories (from a recommended daily or weekly consumption) (**Table S3**). This frequency is noted 0 for no consumption and goes up to 5 for a recommended consumption for each food group presumed to be beneficial (cereals, fruits, vegetables, legumes, olive oil, fish and potatoes). Regarding the other food groups considered as less representative of this dietary pattern (meat, meat products, poultry and dairy products), an inverse scale of the frequency of consumption is used (0 for the highest consumption, up to 5 for the lowest consumptions). Finally, for alcohol, no point is awarded for a consumption of more than 700mL per day or for no consumption, a score of 1 for a consumption of ≥ 600mL and ≤ 700mL per day, a score of 2 for a consumption of ≥ 500mL and < 600mL per day, and so on, with the number of points progressively increasing with the decrease per 100mL of alcohol consumption, up to 5 points awarded for a daily consumption of < 300mL. The obtained MedDietScore summing the values of the 11 food groups indicated the degree of adherence to the MeDi, from 0 to 55 (the highest adherence).

Table S1. Adherence to the Mediterranean Diet according to the MEDI-LITE (0-18) score (Sofi et al.)

**Food groups**	**0 point**	**1 point**	**2 points**
Fruits<br>*1 portion = 150g*	<1 portion/day	[1-1,5] portions/day	>1,5 portions/day
Vegetables<br>*1 portion = 100g*	<1portion/day	[1-2,5] portions/day	>2,5 portions/day
Legumes<br>*1 portion = 70g*	<1 portion par semaine	[1-2] portions par semaine	>2 portions par semaine
Cereals<br>*1 portion = 70g*	<1 portion/day	[1-1,5] portions/day	>1,5 portions/day
Fish<br>*1 portion = 100g*	<1portion par semaine	[1-2,5] portions par semaine	>2,5 portions par semaine
Meats and meats products<br>*1 portion = 80g*	>1,5 portions/day	[1-1,5] portions/day	<1 portion/day
Diary products<br>*1 portion = 180g*	>1,5 portions/day	[1-1,5] portions/day	<1 portion/day
Alcohol<br>*1 alcohol unit = 12g*	>2 alcohol units/day	<1 alcohol unit/day	1-2 alcohol units/day
Olive oil	Occasional use	Frequent use	Regular use

Table S2. Adherence to the Mediterranean Diet according to the Mediterranean Diet Score MDS (0-9) (Trichopoulou et al.)

Food groups	0 point	1 point
Fruits	< gender-specific median	≥ gender-specific median
Vegetables	< gender-specific median	≥ gender-specific median
Cereals	< gender-specific median	≥ gender-specific median
Meats and meat products	> gender-specific median	≤ gender-specific median
Diary product	> gender-specific median	≤ gender-specific median
Alcohol<br>(in g/day)	♂ : <10 and >50<br>♀ : <5 and >25	♂ : [10 ; 50]<br>♀ : [5 ; 25]
Legumes	< gender-specific median	≥ gender-specific median
Fish	< gender-specific median	≥ gender-specific median
Monounsaturated Fatty Acids/Saturated Fatty Acids	< gender-specific median	≥ gender-specific median

Table S3. Adherence to the Mediterranean Diet according to the MedDietScore (0-55) (Panagiotakos et al.)

Food groups	Points by weekly consumption frequency					
Unrefined cereals	Never	]0-6]	]6-12]	]12-18]	]18-32]	>32
	**0**	**1**	**2**	**3**	**4**	**5**
Potatoes	Never	]0-4]	]4-8]	]8-12]	]12-18]	>18
	**0**	**1**	**2**	**3**	**4**	**5**
Fruits	Never	]0-4]	]4 -8]	]8-15]	]15-22]	>22
	**0**	**1**	**2**	**3**	**4**	**5**
Vegetables	Never	]0-6]	]6-12]	]12-20]	]20,5-33]	>33
	**0**	**1**	**2**	**3**	**4**	**5**
Legumes	Never	]0-1]	]1-2]	]2-4]	]4-6]	>6
	**0**	**1**	**2**	**3**	**4**	**5**
Fish	Never	]0-1]	]1-2]	]2-4]	]4-6]	>6
	**0**	**1**	**2**	**3**	**4**	**5**
Red meat and meat products	≤1	]1-3]	]3-5]	]5-7]	]7-10]	>10
	**5**	**4**	**3**	**2**	**1**	**0**
Poultry	≤3	]3-5]	]5-6]	]6-8]	]8-10]	>10
	**5**	**4**	**3**	**2**	**1**	**0**
Diary products	≤10	]10-15]	]15-20]	]20-28]	]28-30]	>30
	**5**	**4**	**3**	**2**	**1**	**0**
Olive oil use	Never	Rarely	<1	[1-3]	]3-5]	Daily
	**0**	**1**	**2**	**3**	**4**	**5**
Alcohol in mL	]0-300[	[300-400[	[400-500[	[500-600[	[600-700]	>700 or 0
(100mL = 1 alcohol unit)	**5**	**4**	**3**	**2**	**1**	**0**

Table S4. Association between Mediterranean Diet adherence and the risk of depressive symptomatology (i.e. CES-D score ≥17 for men and CES-D score ≥23 for women and / or antidepressant treatment), from the 3-City Bordeaux cohort, 2001-2017. (N=1,018).

	**Incident DS /Total**	**OR [95% CI]***	**P-value****
Mediteranean Diet adherence			0.678
- Low adherence	81/257	1	
- Moderate adherence	127/389	0.95 [0.48 ; 1.88]	
- High adherence	113/372	0.76 [0.38 ; 1.53]	

* Random-effect logistic regression model with a random intercept and a random slope adjusted for gender, living condition, tobacco consumption, educational level, regular physical activity, BMI, total energy intake and multimorbidity

** P-value of the log-likelihood ratio test

Low adherence: score 0 to 9

Moderate adherence: score 10 to 11

High adherence: score 12 to 18

References

1. Sofi F, Dinu M, Pagliai G, Marcucci R, Casini A. Validation of a literature-based adherence score to Mediterranean diet: the MEDI-LITE score. International Journal of Food Sciences and Nutrition. 2017 Aug 18;68(6):757-62.

2. Trichopoulou A, Orfanos P, Norat T, Bueno-de-Mesquita B, Ocké MC, Peeters PH, et al. Modified Mediterranean diet and survival: EPIC-elderly prospective cohort study. BMJ. 2005 Apr 30;330(7498):991.

3. Panagiotakos DB, Pitsavos C, Arvaniti F, Stefanadis C. Adherence to the Mediterranean food pattern predicts the prevalence of hypertension, hypercholesterolemia, diabetes and obesity, among healthy adults; the accuracy of the MedDietScore. Preventive Medicine. 2007 Apr 1;44(4):335-40.
